# Inhibition of salt-inducible kinases resolves autoimmune arthritis by promoting macrophage efferocytosis

**DOI:** 10.1038/s41392-025-02381-x

**Published:** 2025-09-12

**Authors:** Mingyu Lee, Min Kyung Kim, Shenzheng Mo, Yoe-Sik Bae, Hong-Hee Kim

**Affiliations:** 1 Department of Health Science and Technology, SAIHST, Sungkyunkwan University, Seoul, Republic of Korea; 2 Department of Cell and Developmental Biology, Dental Research Institute, School of Dentistry, Seoul National University, Seoul, Republic of Korea

**Keywords:** Rheumatic diseases, Prognostic markers, Immunological disorders, Innate immunity, Target identification

**Dear Editor**,

In treating autoimmune diseases like rheumatoid arthritis (RA), the ultimate goals are resolving inflammation and establishing peripheral tolerance.^1^ These processes are closely associated with efferocytosis, the clearance of apoptotic cells and debris by phagocytes in inflamed tissue, which fosters the reprogramming of efferocytic cells to an anti-inflammatory and repair-oriented phenotype while silencing inflammatory signaling cues and antigen presentation.^2^ Thus, efferocytosis-modulatory molecules have emerged as therapeutic targets to boost immune resolution. Salt-inducible kinases (SIKs) regulate intracellular signaling and transcriptional machinery.^3^ However, the roles of SIKs in efferocytosis and RA immune responses have not been investigated.

In analyses of a public dataset (GSE13071) from synovial tissue (ST) of collagen-induced arthritis (CIA) mice, we found that the levels of SIK1/3 and the macrophage marker F4/80 gradually increased with the severity of the disease (Fig. 1a). With a hypothesis that SIK1/3 upregulation might exacerbate RA, we tested a pan-SIK inhibitor, YKL-05-099, on CIA. Administrating YKL-05-099, after 2^nd^ collagen immunization, significantly reduced joint and bone destruction and immune cell infiltration in the paws, preserving clear territories of synovial tissues and cartilage (Fig. 1a). As CIA became severe, the levels of Sgk1 and CD36, efferocytic function indicators,^2^ decreased while AnnexinV, the apoptotic cell marker,^2^ increased in ST (Fig. 1a). YKL-05-099 treatment greatly reduced TUNEL^+^ apoptotic cells (ACs) and increased the contact between macrophage and AC in ST of CIA mice (Fig. 1a). These results demonstrate that SIKs inhibition ameliorates RA symptoms and also imply that this therapeutic effect may be attributable to AC removal by macrophages.

**Fig. 1:**
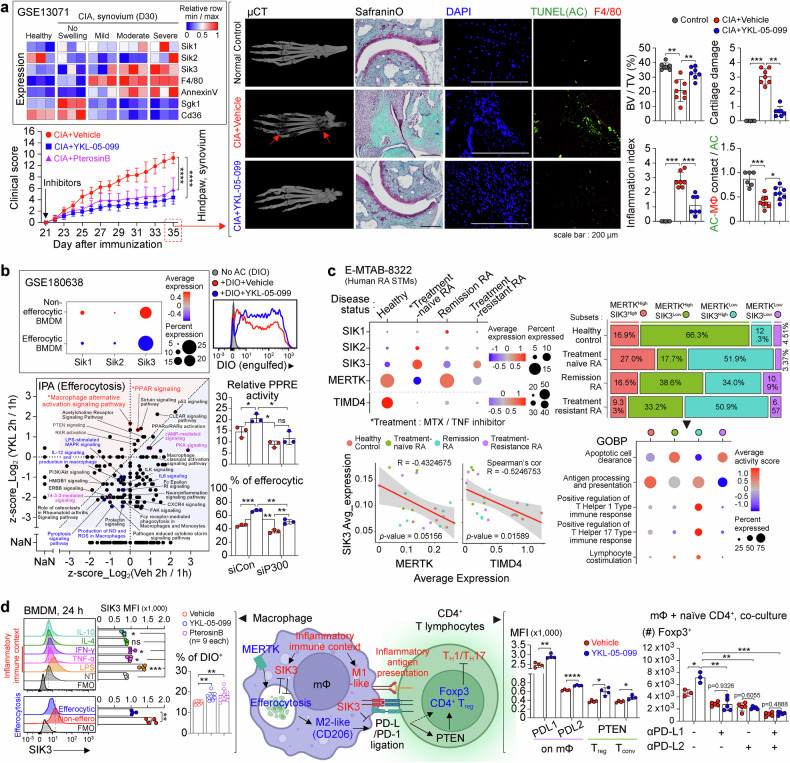
Inhibition of SIKs promotes macrophage efferocytosis and regulates CD4^+^ T cell immune responses. **a** Gene expression patterns of SIKs and efferocytosis-related transcripts in synovial tissue during collagen-induced arthritis (CIA) progression (GSE13071) and clinical scores of female CIA mice injected with vehicle (*n* = 9), a pan SIK inhibitor YKL-05-099 (20 mg/kg, *n* = 10), or a SIK3-selective inhibitor Pterosin B (20 mg/kg, *n* = 10). Hindpaws of normal DBA1/J or CIA mice were fixed and scanned with μCT, followed by immunohistochemistry to identify cartilage damage (Safranin O, violet indicates cartilage) and inflammation score (cell infiltration). Arrows indicate the hollow region due to osteolysis. Immunofluorescence analyses for the presence of apoptotic cells (TUNEL^+^) and F4/80^+^ macrophages among the infiltrated cells (DAPI^+^). **b** Expression of SIKs in non-efferocytic and efferocytic BMDMs analyzed using scRNAseq data (GSE180638), and experimental comparisons of efferocytic efficiency (DIO engulfment) over 24 h in BMDMs treated with YKL-05-099 (1 μM). An ingenuity pathway analysis (QIAGEN, IPA) on the bulk mRNAseq dataset obtained from efferocytic BMDMs treated with YKL-05-099. NaN indicates non-available number. Luciferase reporter activities of nuclear peroxisome proliferator-response elements (PPREs) and efferocytic efficacy in p300-knockdowned BMDMs. **c** Relative average expression of SIKs, MERTK, and TIMD4, and the frequency of expressing cells according to disease status. MTX, methotrexate. The relationship between SIK3 expression and MERTK or TIMD4 expression in RA patients STMs. Percentages of STM subsets, classified based on SIK3 and MERTK expressions, in RA patients. Gene ontology (GO) analysis related to biological process (BP) of the STM subsets. **d** SIK3 expression in BMDMs challenged with each cytokine (10 ng/ml) or LPS (100 ng/ml) for 24 h. SIK3 levels in BMDMs alone or co-cultured with lipophilic dye-labeled apoptotic cells. Frequency of efferocytic macrophages (DIO^+^) in BMDMs treated with YKL-05-099 (1 μM) or Pterosin B (20 μM) for 4 h. Expression of PD-L1/L2 in BMDMs challenged with vehicle or YKL-05-099 for 24 h and PTEN level of CD4^+^ T cells in αCD3e-activated splenocytes treated with vehicle or YKL-05-099 for 72–96 h. Number of CD4^+^ Foxp3^+^ T_reg_ in macrophage-naïve CD4^+^ T cell cocultures, and effects of PD-L1 and PD-L2 blocking antibodies on YKL-05-099-mediated T_reg_ generation. The image of (**d**) in the middle was created with the assistance of BioRender (https://www.biorender.com/). Data are expressed as the mean ± S.D. and *p*-values were calculated using the unpaired parametric Welch’s corrected *t*-test (two-tailed). (*P < 0.05, **P < 0.01, ***P < 0.001, ****P < 0.0001)

With the possibility of efferocytosis regulation by SIKs in RA, we next analyzed SIK levels in efferocytic and non-efferocytic macrophages. scRNAseq data (GSE180638) revealed that the expression of SIKs, especially SIK3, is markedly lower in efferocytic bone marrow-derived macrophages (BMDMs) than in non-efferocytic BMDMs. In our experiments with DIO-labeled ACs, YKL-05-099 significantly enhanced efferocytosis by BMDMs (Fig. 1b). To explore how SIK inhibition promotes efferocytosis, we conducted bulk mRNA-seq of efferocytic BMDMs and performed in silico analyses with the Ingenuity Pathway Analysis (IPA). IPA predicted significant changes in PKA-, cAMP-, and 14-3-3-mediated signaling pathway activities at an early stage of efferocytosis (Fig. 1b). These pathways have been implicated in inhibition of SIKs.^3^ Altogether, these findings suggest that reduction in SIK activity during efferocytosis may be crucial for the progress of efferocytosis. IPA also predicted the stimulation of macrophage alternative activation (M2) and PPARγ signaling pathways by YKL-05-099 (Fig. 1b). In later stages of M2 polarization, PPARγ-RXR heterodimers act as epigenomic regulators, recruiting histone acetyltransferase p300.^4^ PPARγ-mediated transcriptional activation facilitates the induction of efferocytic receptors and bridging surface molecules.^2^ PPARγ also help controlling metabolic stress in efferocytic cells and induces progressive M2 polarization.^2^ We found that SIK inhibition increased early PPARγ transcriptional activity (PPRE) in BMDMs. Moreover, p300 knockdown nullified the effects of YKL-05-099 on PPRE activation and efferocytosis stimulation (Fig. 1b). Collectively, these results indicate that SIK inhibition promotes macrophage efferocytosis via a p300-PPARγ-dependent pathway.

To delve deeply into the relationship between SIK and efferocytosis in the context of RA pathogenesis, we next analyzed scRNA-seq data (E-MTAB-8322) of ST macrophages (STMs) from RA patients. The expression of SIK3, but not that of SIK1/2, was distinct and remarkable when categorized according to the disease state of RA. SIK3 expression showed a significant negative relationship with MERTK and TIMD4 efferocytosis receptors (Fig. 1c). Comparing STM subgroups based on SIK3 and MERTK expression, the MERTK^High^SIK3^Low^ STMs were prominent in healthy individuals. In untreated RA patients, MERTK^High^SIK3^Low^ STMs markedly reduced whereas MERTK^High^SIK3^High^ and MERTK^Low^SIK3^High^ STMs increased. These SIK3^High^ population diminished in patients under remission following RA treatments but MERTK^Low^SIK3^High^ persisted in treatment-resistant patients. SIK3^Low^ STMs showed higher AC clearance and lower antigen presentation activities in gene ontology. Further analyses revealed a transition from MERTK^High^SIK3^Low^ to MERTK^High^SIK3^High^ and then to MERTK^Low^SIK3^High^, which was associated with T cell education for T_H_17/T_H_1 immune responses, during RA progression (Fig. 1c). These findings suggest that the increase in SIK3 impairs efferocytosis and contributes to the shift of macrophage fate towards the activation of antigen presentation related to autoimmune responses in RA. In support of the crucial role of SIK3 in RA, Pterosin B, a selective SIK3 inhibitor, reduced arthritis in CIA mice (Fig. 1a).

Macrophage efferocytosis is interconnected with M2 polarization and T_reg_ expansion for peripheral resolution. In contrast, exposure to pathogen-derived molecules or inflammatory signaling cues during efferocytosis can impair T_reg_ expansion and promote T_H_17 polarization.^2^ Therefore, we next explored the effect of inflammatory stimuli on SIK3 expression in BMDMs. TNFα, IFNγ, and LPS increased SIK3, whereas efferocytosis induction with ACs decreased SIK3 in BMDMs. Furthermore, Pterosin B enhanced efferocytosis (Fig. 1d). Our IPA analysis also indicated activation of M2 pathway and suppression of inflammatory responses by SIK inhibition in efferocytic BMDMs (Fig. 1b). Decreases in M1 polarization and inflammatory cytokines by SIK inhibition were also observed. While patients who respond well to treatment often show increased T_reg_ levels, the mechanism of their induction remains elusive.^1^ PD-1 signaling triggered by PD-L1/2 has been reported to upregulate Foxp3 via PTEN.^5^ We found that PD-L1/2 on BMDMs were significantly elevated by YKL-05-099 (Fig. 1d). The expression of PTEN in CD4^+^ T cells of splenocyte cultures was also increased by SIK inhibition. Furthermore, in macrophage-CD4^+^ T cell co-cultures, the Foxp3^+^ T_reg_ population was increased by YKL-05-099. Inclusion of anti-PD-L1/2 antibodies blocked the effect of T_reg_ induction by YKL-05-099 in the co-cultures (Fig. 1d). These findings indicate that SIK inhibition stimulates M2 polarization and PD-L1/2 expressions in macrophages, which promote T_reg_ differentiation through PD-1 ligation and subsequent PTEN activation. Thus, the suppression of SIK3 may explain the mechanism for the increase of T_reg_ cells in RA patients in remission state.

Collectively, this study discovered for the first time that SIKs, especially SIK3, play a crucial role in inhibiting macrophage efferocytosis. We also demonstrated that SIK inhibition alleviates bone and cartilage destruction as well as synovial inflammation in CIA. In addition to promoting efferocytosis, SIK inhibition leads to M2 polarization and T_reg_ differentiation. These combinatorial effects may result in immune resolution and peripheral tolerance, thereby achieving ultimate remission status in RA patients. These features may suit SIKs as promising targets in the development of therapeutics for RA and other chronic autoimmune diseases.

## Supplementary information

Supplementary materials for Inhibition of salt-inducible kinases resolves autoimmune arthritis by promoting macrophage efferocytosis

## Data Availability

All materials, antibodies, and mice used in this study are commercially available, and this study did not generate any new unique reagents and methods. The data supporting this research are available within the paper or derived by re-analyzing published data in open databases, as indicated in the figures or figure legends. Raw and processed data of bulk mRNAseq have been deposited in an open-data base (BioProject) with the dataset identifier as Project PRJCA035177 (https://ngdc.cncb.ac.cn/bioproject/browse/PRJCA035177). Source raw data and procedures of R analysis are available from the corresponding author upon reasonable request.
