# Supplementary material for: Inhibition of salt-inducible kinases resolves autoimmune arthritis by promoting macrophage efferocytosis
Source: Signal Transduct Target Ther. 2025 Sep 12;10:293. doi: 10.1038/s41392-025-02381-x (PMC12426215; doi:10.1038/s41392-025-02381-x)
Supplement: Supplementary file 1 — Supplementary materials for Inhibition of salt-inducible kinases resolves autoimmune arthritis by promoting macrophage efferocytosis [file 41392_2025_2381_MOESM1_ESM.docx]

Supplementary materials for

**Inhibition of salt-inducible kinases resolves autoimmune arthritis by promoting macrophage efferocytosis**

Mingyu Lee^1,§^, Min Kyung Kim^2,§^, Shenzheng Mo^2^, Yoe-Sik Bae^1,*^, and Hong-Hee Kim^2,*^

^*^Correspondence to: hhbkim@snu.ac.kr, yoesik@skku.edu.

Lead contact : Hong-Hee Kim.

This supplementary material file includes:

Materials and Methods

**Materials and Methods**

**Collagen-induced arthritis (CIA).** 8-10-week-old male or female DBA1/J mice were allowed to adapt for 1 weak before experiments. To establish CIA model, mice were intradermally immunized in the tail with bovine type II collagen (Chondrex, 20022) emulsified in complete freund’s adjuvant (Chondrex, 7001) on day 0 and incomplete freund’s adjuvant (Chondrex, 7002) on day 21. After 2^nd^ immunization, mice were randomly assigned to experimental groups and YKL-05-099 (20 mg/kg) or Pterosin B (20 mg/kg) were administered daily until sacrifice. SIK inhibitor YKL-05-099 and Pterosin B were purchased from MedChemExpress, and is diluted in 5% Solutol HS15, 5% N-methyl-2-pyrrolidinone, and 90% saline.

**Analysis of hindpaws.** Hindpaws of sacrificed mice were analyzed using μCT scanner (Skyscan). Regions near the proximal interphalangeal joints of digits were scanned (thresholds range: 70~255) and bone volume per tissue volume (BV/TV) was obtained from reconstituted images using CT program software. Hindpaws were fixed in 4% paraformaldehyde and decalcified for 4 weeks in 12% EDTA in PBS (pH 7.4), then prepared as paraffin-embedded sections (5 μm). The sections were deparaffinized in xylene and rehydrated in ethanol and stained with safranin O. For immunofluorescent staining, sections were heated in 10 mM of sodium citrate (pH 6.0) overnight at 65˚C for antigen retrieval and then incubated for 20 minutes in 3% H_2_O_2_ in methanol to block endogenous peroxidase activity. Processed sections were incubated with anti-F4/80 antibody (Abcam, ab111101) overnight at 4˚C, and then incubated with Alexa 647-conjugated anti-rabbit IgG (Thermo Scientific, A32731). Subsequently, sections were stained with TUNEL Assay kit (Abcam, ab166108), followed by DAPI staining.

**Analysis of macrophages.** Bone marrow cells were isolated by flushing bone marrow of tibiae and femurs and cultured overnight in α-MEM supplemented with 10% fetal bovine serum. Non-adherent cells were collected and cultured with 30 ng/ml of mM-CSF on petri dishes. After 4-5 days of culture, adherent cells were harvested and designated as BMDMs. For co-culture experiments, splenic F4/80^+^ macrophages and naïve CD4^+^ T cells were isolated with magnetic bead-based selection kits (Invitrogen, F4/80, 8802-6863-74; naïve CD4, 8804-6824-74) according to manufacturer’s instruction. BMDMs or splenic macrophage (5×10^4^) were co-cultured with naïve CD4^+^ T cells (2.5×10^5^) in the presence of soluble anti-CD3ε (1 μg/ml) for 72~ 96 hours. For investigation of efferocytic capacity of macrophages, human Jurkat T cells were irradiated with a 254-nm UV lamp for generation of apoptotic cells (ACs). ACs were validated with annexin V and PI staining using flow cytometry. For labelling ACs, Vybrant^TM^ Cell-labeling solution (Invitrogen, V22886) was used according to the manufacturer’s instruction. BMDMs were co-cultured with labeled ACs at a ratio of 1:5~1:10. Recombinant murine M-CSF, TNF-α, IFN-γ, IL-4, and IL-10 were purchased from PeproTech (USA). LPS was purchased from sigma aldrich.

**Flow cytometry.** Cells were washed with and prepared in PBS, then incubated with fixable viability dye eFluor^TM^ 780 (Invitrogen, 65-0865) in PBS, followed by blocking with anti-CD16/32 (Invitrogen, 14-0161) in FACS buffer (0.5% BSA in PBS). The cells were washed and incubated with fluorochrome-conjugated surface and intracellular antibodies. For intracellular protein staining, surface-stained cells were fixed and permeabilized with Foxp3 TF fixation/permeabilization buffer (Invitrogen, 00-5521-00). For staining antibodies that are not conjugated with fluorochromes, anti-rabbit IgG (H+L) 2^nd^ antibodies conjugated to AF488 (Invitrogen, A011934) or 647 (Invitrogen, A21240) were used. Suspended cells were analyzed with flow cytometers (FACSCanto^TM^, BD) and evaluated with the FlowJo program (v10.10, BD Biosciences). Antibodies used for flow cytometry were purchased from Invitrogen; anti-F4/80 (BM8), anti-CD4 (RM4-5), anti-PD-L1 (MIH5), anti-PD-L2 (TY25), anti-MERTK (DS5MMER), and anti-Foxp3 (FJK-16s). Antibody for anti-SIK3 staining was from abcam (ab255701) and antibody for PTEN was from R&D (IC847G).

**PPRE activity** **assay.** To examine transcriptional activity, macrophages were transfected with PPRE-luc, pCMV-Renilla constructs using lipofectamine 3000 (Invitrogen, L3000001) according to the vendor’s protocol. The transfected BMDMs were co-cultured with ACs and then lysed for luciferase reporter assay using the Dual-Luciferase Reporter Assay System kit (Promega) according to the manufacturer’s instruction. Luciferase activity was measured using a Synergy H1 multi-mode microplate reader (Bio-tek). PPRE-luciferase activity was normalized to the Renilla luciferase activity in each sample.

**Bulk mRNAseq analysis of efferocytic BMDMs.** Macrophages treated with either vehicle or YKL-05-099 were cultured with ACs, and total RNA was isolated from individual samples using TRIzol. The quality of RNA was assessed using the Agilent 2100 bioanalyzer with the RNA 6000 Nano kit (Agilent Technologies), and the eluted RNA was quantified using ND-2000 Spectrophotometer (Thermo Scientific). All RNA samples exhibited high quality (RNA integrity number equivalent > 7.0 and OD260/280 > 1.80) and quantity (RNA concentration > 70 ng/μl). Sequencing was performed on the NextSeq 500 (Illumina) platform according to the manufacturer’s instructions by ebiogen, Inc. (Seoul, Korea). For Ingenuity Pathway Analysis (IPA), the fold changes and p-values from the bulk mRNA-seq data of efferocytic BMDMs were used in IPA programs. Subsequently, pathway analysis was conducted using a threshold of p-value less than 0.05.

**Analyses of scRNA sequencing data from open database.** The analysis of scRNA-seq data was conducted according to Seurat’s standard workflow and standard guidelines (5.1.0) (<https://doi.org/10.1038/s41587-023-01767-y>), following the information provided in the reference paper [CIA ST, GSE13071 (<https://doi.org/https://doi.org/10.1038/mt.2009.182>); RA patients STM, E-TAB-8322 (<https://doi.org/10.1038/s41591-020-0939-8>); Efferocytic/non-efferocytic BMDMs, GSE180638 (<https://doi.org/10.3390/cells11233712>)] for analysis of SIK expressions. Activity of specific features or the average expression and expressing percentage of genes were investigated using the ‘DotPlot’ and ‘AddModuleScore’ functions. Relationships between genes were compared using Spearman correlation. To analyze the trajectory, the expression matrix, metadata, and gene metadata were extracted from the Seurat object and converted into a Monocle3 object following Monocle3’s guidelines and standard procedures (<https://doi.org/10.1038/s41586-019-0969-x>). The starting point of the trajectory was selected based on changes in different experimental conditions and disease states.

**Statistical analysis.** Data were evaluated using GraphPad prism 10.5.0 software (<https://www.graphpad.com/>) and mainly expressed as the mean ± standard deviation (S.D.). Relative row maximum (1) and minimum value (0) were automatically calculated by Morpheus (<https://software.broadinstitute.org/morpheus>). z-score was calculated by subtracting the mean from the value and then dividing it by the S.D. of the data. *p*-values were calculated using the unpaired parametric Welch’s corrected t-test (two-tailed) and a *p*-value of lower than 0.05 was considered significant. **P* < 0.05, ***P* < 0.01, ****P* < 0.001, *****P* < 0.0001.
